# *Sulfolobus* – A Potential Key Organism in Future Biotechnology

**DOI:** 10.3389/fmicb.2017.02474

**Published:** 2017-12-12

**Authors:** Julian Quehenberger, Lu Shen, Sonja-Verena Albers, Bettina Siebers, Oliver Spadiut

**Affiliations:** ^1^Research Division Biochemical Engineering, Faculty of Technical Chemistry, Institute of Chemical, Environmental and Biological Engineering, Vienna University of Technology, Vienna, Austria; ^2^Department of Molecular Enzyme Technology and Biochemistry, Faculty of Chemistry – Biofilm Centre, University of Duisburg-Essen, Essen, Germany; ^3^Molecular Biology of Archaea, Institute of Biology II-Microbiology, Faculty of Biology, University of Freiburg, Freiburg im Breisgau, Germany

**Keywords:** *Sulfolobus*, biotechnology, thermophile, acidophile, bioprocessing, biorefinery

## Abstract

Extremophilic organisms represent a potentially valuable resource for the development of novel bioprocesses. They can act as a source for stable enzymes and unique biomaterials. Extremophiles are capable of carrying out microbial processes and biotransformations under extremely hostile conditions. Extreme thermoacidophilic members of the well-characterized genus *Sulfolobus* are outstanding in their ability to thrive at both high temperatures and low pH. This review gives an overview of the biological system *Sulfolobus* including its central carbon metabolism and the development of tools for its genetic manipulation. We highlight findings of commercial relevance and focus on potential industrial applications. Finally, the current state of bioreactor cultivations is summarized and we discuss the use of *Sulfolobus* species in biorefinery applications.

## Introduction

Thermophiles gain increasing attention in biotechnological applications due to their potential to expand the thermal range of industrial biotechnology and their unique metabolic capabilities (Littlechild, 2015; Zeldes et al., 2015; Beeler and Singh, 2016; Donati et al., 2016; Basen and Müller, 2017; Straub et al., 2017). In this review, we focus on the well-characterized members of the phylum Crenarchaeota, the extreme thermoacidophilic Archaea belonging to the genus *Sulfolobus*. Natural habitats of these organisms are solfataric fields all around the world, including the United States, Costa Rica, Mexico, Russia, Japan, China, New Zealand, Germany, Italy, and Iceland. The outstanding characteristic of these organisms, which have been investigated since the 1970s (**Supplementary Table S1**), is their ability to thrive at extremely low pH and high temperature, unprecedented in Eukaryotes and Bacteria.

Since *Sulfolobus* spp. can be grown and manipulated under laboratory conditions, they are popular model organisms to study Archaea. Research has been focused on their biology and physiology. Currently, genomics (Bell et al., 2002; Dai et al., 2016), proteomics (Chong and Wright, 2005; Ellen et al., 2010; Pham et al., 2010; Kort et al., 2013), metabolomics (Ulas et al., 2012; Bräsen et al., 2014), composition and function of the archaeal membrane (Albers and Meyer, 2011) and the archaellum (Albers and Jarrell, 2015), as well as interaction with archaeal viruses (Prangishvili et al., 2006) are important fields of research. Nevertheless, there is also growing interest in the utilization of this genus in biotechnological applications and the development of engineered strains to exploit the organisms’ unique characteristics. *Sulfolobus* spp. are a source of unique enzymes (Littlechild, 2015), biomaterials (Benvegnu et al., 2009; Besse et al., 2015), and metabolic pathways (Bräsen et al., 2014). As most prominent examples, the branched Entner–Doudoroff (ED) pathway (Kouril et al., 2013b) as well as Weimberg and Dahms pathways for the degradation of hexoses and pentoses (Nunn et al., 2010) should be named. These diverse catabolic pathways present a promising field for the exploitation of novel products (Ahmed et al., 2005; Siebers and Schönheit, 2005).

**FIGURE 1:**
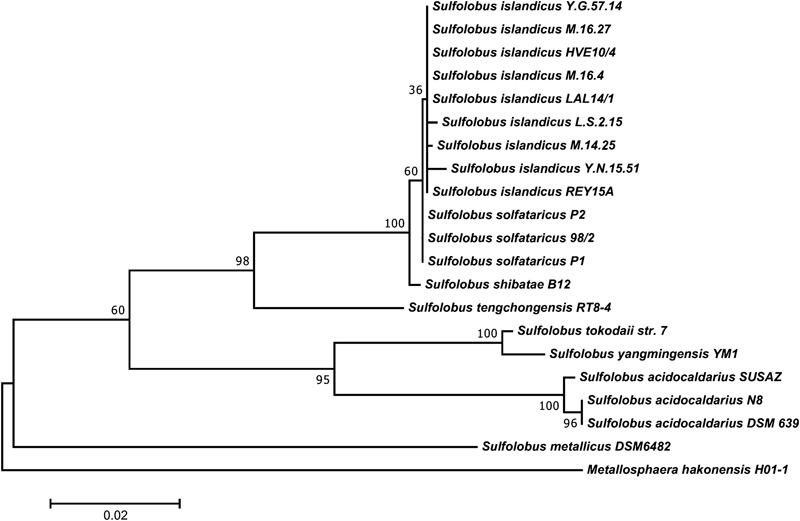
Phylogenetic tree of the genus *Sulfolobus* based on all publicly available 16S rDNA sequences of acknowledged species. The tree was constructed with MEGA 7.0 using the maximum-likelihood method after automated alignment with clustalX2 and manual correction with GeneDoc. The percentages of replicate trees in which the associated taxa clustered together in the bootstrap test (1000 replicates) are shown next to the branches. The tree is drawn to scale, with branch lengths measured in the number of substitutions per site. *Metallosphaera hakonensis* H01-1 was used as out-group.

Among the eight *Sulfolobus* species established in the literature, *S. islandicus, S. solfataricus*, and *S. acidocaldarius* are by far the best described members of the genus. While *S. islandicus* is used as a model organism for comparative genomics and genetics (Reno et al., 2009) and for host–virus interactions (Held and Whitaker, 2009), no type strain has been designated and strains are not commercially available yet. *S. solfataricus* is the metabolically most diverse species and many catabolic enzymes have been investigated in detail (Bräsen et al., 2014). Unfortunately, this diversity comes along with a significant genetic instability caused by the presence of several hundred mobile elements identified in its genome (Brügger et al., 2002). By contrast, the genome of *S. acidocaldarius* is much more stable (Chen et al., 2005). This makes *S. acidocaldarius* interesting for industrial applications, where strain stability is of utmost importance. A phylogenetic tree of the genus *Sulfolobus* is shown in **Figure 1**.

In this review, we give an overview of the current state of knowledge on carbon metabolism, genetic tools, and fermentation techniques of *Sulfolobus* spp., describe relevant products, and discuss potential future applications of this genus.

## Central Carbon Metabolism

**FIGURE 2:**
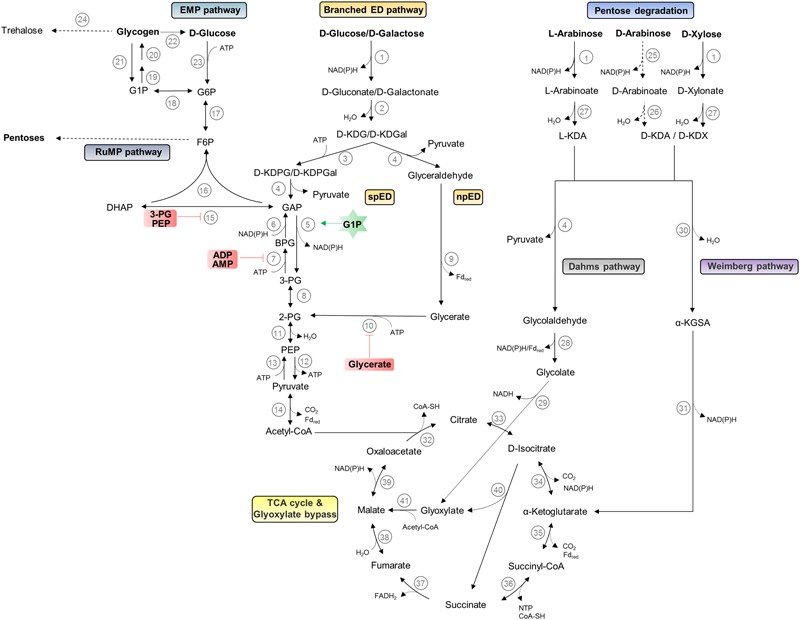
Central carbohydrate metabolism in *Sulfolobus* spp. The pathways for hexose and pentose degradation as well as glycogen, trehalose, and pentose formation are shown. D-arabinose (dashed lines) can only be utilized as carbon source by *S. solfataricus* and not by *S. acidocaldarius*. The current understanding of regulation by effectors is indicated by green stars and red boxes for activator and inhibitors, respectively. Enzymes catalyzing different reactions are depicted as numbers: (1) glucose dehydrogenase (broad substrate specificity); (2) gluconate dehydratase; (3) 2-keto-3-deoxygluconate kinase; (4) 2-keto-3-deoxy-(6-phospho) gluconate aldolase (broad substrate specificity); (5) non-phosphorylating glyceraldehyde-3-phosphate dehydrogenase; (6) glyceraldehyde-3-phosphate dehydrogenase; (7) phosphoglycerate kinase; (8) phosphoglycerate mutase; (9) glyceraldehyde:ferredoxin oxidoreductase; (10) glycerate kinase; (11) enolase; (12) pyruvate kinase; (13) phosphoenolpyruvate synthetase; (14) pyruvate:ferredoxin oxidoreductase; (15) triosephosphate isomerase; (16) fructose-1,-6-bisphosphate aldolase/phosphatase; (17) phosphoglucose/phosphomannose isomerase; (18) phosphoglucomutase/phosphomannomutase; (19) NTP-glucose-1-phosphate uridylyltransferase; (20) glycogen synthase; (21) glycogen phosphorylase; (22) glucan-1,4-α-glucosidase; (23) hexokinase; (24) maltooligosyltrehalose synthase/maltooligosyltrehalose trehalohydrolase; (25) D-arabinose dehydrogenase; (26) D-arabinoate dehydratase; (27) L-arabinoate/D-xylonate dehydratase; (28) glycolaldehyde dehydrogenase/glycolaldehyde:ferredoxin oxidoreductase; (29) glycolate dehydrogenase; (30) 2-keto-3-deoxy-arabinoate/xylonate dehydratase; (31) α-ketoglutarate semi-aldehyde dehydrogenase; (32) citrate synthase; (33) aconitase; (34) isocitrate dehydrogenase; (35) α-ketoglutarate:ferredoxin oxidoreductase; (36) succinyl-CoA synthetase; (37) succinate dehydrogenase; (38) fumarase; (39) malate dehydrogenase; (40) isocitrate lyase; (41) malate synthetase. EMP, Embden–Meyerhof–Parnas; ED, Entner–Doudoroff; spED, semi-phosphorylative ED; npED, non-phosphorylative ED; RuMP, reversed ribulose monophosphate; TCA, tricarboxylic acid; G1P, glucose 1-phosphate; G6P, glucose 6-phosphate; F6P, fructose 6-phosphate; DHAP, dihydroxyacetone phosphate; GAP, glyceraldehyde 3-phosphate; BPG, 1,3-bisphosphoglycerate; 3-PG, 3-phosphoglycerate; 2-PG, 2-phosphoglycerate; PEP, phosphoenolpyruvate; D-KDG, 2-keto-3-deoxy-D-gluconate; D-KDGal, 2-keto-3-deoxy-D-galactonate; D-KDPG, 2-keto-3-deoxy-6-phosphate-D-gluconate; D-KDPGal, 2-keto-3-deoxy-6-phosphate-D-galactonate; L-KDA, 2-keto-3-deoxy-L-arabinoate; D-KDA, 2-keto-3-deoxy-D-arabinoate; D-KDX, 2-keto-3-deoxy-D-xylonate; α-KGSA, α-ketoglutarate semi-aldehyde.

*Sulfolobus* spp. thrive at pH 2–3 and temperatures around 75–80°C. They are characterized by a chemoorganoheterotrophic lifestyle; however, chemolithoautotrophic growth using sulfur oxidation has been reported for some species (Huber et al., 1992; Schönheit and Schäfer, 1995). All *Sulfolobus* species exhibit an aerobic lifestyle and for *S. solfataricus* P2, a preferred growth at lower oxygen concentrations was reported (Grogan, 1989; Simon et al., 2009). The different *Sulfolobus* strains differ significantly in their metabolic potential. *S. solfataricus* possesses a broad substrate specificity and uses various sugars such as polysaccharides (e.g., cellulose, starch, dextrin), disaccharides (e.g., maltose and sucrose), hexoses (e.g., D-glucose, D-galactose, D-mannose, and L-fucose), pentoses (e.g., D-arabinose, L-arabinose, D-xylose), aldehydes, alcohols (e.g., ethanol, phenol), sugar acids as well as tryptone, peptides, and amino acids as carbon source (Grogan, 1989; Izzo et al., 2005; Brouns et al., 2006; Joshua et al., 2011; Comte et al., 2013; Wolf et al., 2016; Stark et al., 2017). For *S. solfataricus*, a genome scale model comprising 718 metabolic and 58 transport/exchange reactions and 705 metabolites was used to simulate growth on 35 different carbon sources (Ulas et al., 2012). While no such modeled data are published for *S. acidocaldarius*, traditional growth experiments suggest that this species is well adapted to proteolytic growth and can utilize only few other carbon sources such as dextrin, sucrose, D-glucose, D-xylose, and L-arabinose (Grogan, 1989; Joshua et al., 2011). The differences in the metabolic potential are also reflected by the respective genome size of 2.99 Mbp including 200 IS elements for *S. solfataricus* (She et al., 2001) and of 2.23 Mbp for *S. acidocaldarius* (Chen et al., 2005). In the following paragraphs, we sum up the knowledge on the central carbohydrate metabolism and give an illustration of these pathways in **Figure 2** (hexose and pentose degradation as well as glycogen, trehalose, and pentose formation).

Like most aerobic bacteria *Sulfolobus* spp. rely on the ED pathway for carbon degradation; however, in contrast to the classical pathway found, for example, in *Pseudomonas* species (Entner and Doudoroff, 1952), the archaeal pathway is branched and omits the initial phosphorylation of D-glucose. Instead, the sugar is directly oxidized to D-gluconate and dehydrated to 2-keto-3-deoxygluconate (KDG) as the characteristic intermediate of the pathway. In *S. solfataricus* KDG is either directly cleaved by the bifunctional aldolase to pyruvate and glyceraldehyde in the non-phosphorylative (np) branch of the ED pathway or first phosphorylated to 2-keto-3-deoxy-6-phosphogluconate (KDPG) and cleaved to pyruvate and glyceraldehyde 3-phosphate (GAP) in the semi-phosphorylated (sp) branch of the ED pathway. In the npED branch, glyceraldehyde is further oxidized and phosphorylated by glyceraldehyde:ferredoxin oxidoreductase and glycerate kinase to 2-phophoglycerate, which enters the lower shunt of the Embden–Meyerhof–Parnas (EMP) pathway (Ahmed et al., 2005). In the spED, GAP is oxidized to 3-phosphoglycerate by a non-phosphorylating GAP dehydrogenase (GAPN), activated by glucose 1-phophate, replacing the classical GAP dehydrogenase (GAPDH) and phosphoglycerate kinase (PGK) couple (GAPDH/PGK). The pathway in *S. solfataricus* is promiscuous for D-glucose and D-galactose (Lamble et al., 2005). Metabolome analysis of the KDG kinase deletion strain revealed a major function of the spED pathway in providing GAP for gluconeogenesis (Kouril et al., 2013b).

Pyruvate is further oxidatively decarboxylated to acetyl-CoA via the pyruvate:ferredoxin oxidoreductase; the classical pyruvate dehydrogenase complex is absent in Archaea. Acetyl CoA enters the oxidative citric acid cycle and is finally completely oxidized to two molecules of CO_2_. The substitution of the catabolic GAPDH and PGK couple by GAPN results in no net gain of ATP in the branched ED pathway. Only in the citric acid cycle, the succinyl-CoA synthetase is supposed to provide nucleoside triphosphate (NTP) by substrate level phosphorylation. Therefore, the major energy gain comes from aerobic respiration. The respiratory chain in several members of the Sulfolobales has been studied, and in *S. solfataricus* as well as in *S. acidocaldarius*, a branched electron transport chain with three terminal oxidases was reported (Schafer et al., 1999; Auernik and Kelly, 2008). For *S. solfataricus* the regulation at transcriptome level in response to different oxygen concentrations was demonstrated (Simon et al., 2009).

In *Sulfolobus* spp., the EMP pathway is only used for gluconeogenesis, although for glycolysis only a functional phosphofructokinase is missing (Kouril et al., 2013a). As key enzymes, especially the classical GAPDH and PGK are only active in the gluconeogenic direction. Further on, a bifunctional, gluconeogenic fructose bisphosphate aldolase/phosphatase (FBPA/ase) catalyzes the one-step formation of fructose 6-phosphate from GAP and dihydroxyacetone phosphate (Say and Fuchs, 2010; Kouril et al., 2013a; Bräsen et al., 2014). Glycogen is formed as carbon storage compound (König et al., 1982) and as source for trehalose formation via the TreY/TreZ pathway [i.e., maltooligosyltrehalose synthase and maltooligosyltrehalose trehalohydrolase (Maruta et al., 1996)]. Trehalose is the only compatible solute reported so far in *Sulfolobus* spp.

Thus, like in all Archaea, the central carbohydrate metabolism in *Sulfolobus* spp. is characterized by unusual pathways and enzymes that – moreover – also confer unique regulatory properties. In contrast to the classical bacterial and eukaryotic EMP pathway, the regulation is established at the level of triose phosphates, which seems to be a general feature in (hyper)thermophilic Archaea with optimal growth close to 80°C. Triose phosphates are labile at high temperatures and it was shown that the thermal degradation of these pathway intermediates is a crucial bottleneck for efficient substrate conversion (Kouril et al., 2013a).

In addition, the upper part of the EMP pathway seems to play an important function for pentose generation. In *Sulfolobus* species, as in most Archaea, the classical pentose phosphate pathway is absent and pentoses are formed from fructose 6-phosphate via the reversed ribulose monophosphate pathway (RuMP) (Soderberg, 2005). The RuMP pathway was previously reported as formaldehyde fixation pathway in methylotrophic bacteria.

Pentose degradation has been studied in *S. solfataricus* and *S. acidocaldarius*. For *S. solfataricus* the D-arabinose degradation was resolved and an oxidative pathway with formation of α-ketoglutarate, which directly enters the citric acid cycle, was demonstrated (Brouns et al., 2006). Later studies revealed that the transporter and degradation pathway is partially promiscuous for L-fucose utilization (Wolf et al., 2016). The D-arabinose and D-xylose pathway merge at the identical intermediates 2-keto-3-deoxy-D-arabionoate (D-KDA) and 2-keto-3-deoxy-D-xylonate (D-KDX). For D-xylose degradation, a branched pathway with an aldolase-dependent branch forming pyruvate and finally glyoxylate (Dahms pathway), which enters the glyoxylate bypass, and an aldolase-independent branch forming the citric acid cycle intermediate α-ketoglutarate (Weimberg pathway) were proposed for *S. solfataricus* (Nunn et al., 2010). Important for cellulosic biomass conversion the absence of diauxic growth on D-glucose and D-xylose was reported for *S. acidocaldarius* (Joshua et al., 2011).

In general, the availability of genome scale models, functional genomics, and systems biology approaches for *Sulfolobales* under different stress and growth conditions in combination with biochemical and genetic studies enabled an in depth insight into metabolism and cellular processes [e.g., growth on L-fucose and casamino acids compared to D-glucose in *S. solfataricus* (Wolf et al., 2016; Stark et al., 2017)]. The established knowledge forms an important prerequisite for the establishment of *Sulfolobus* spp. as thermoacidophilic, archaeal platform organisms using metabolic engineering, and synthetic biology approaches for future biotechnological applications.

## Genetic Tools

The lack of genetic tools has been a major drawback for the establishment of archaeal model organisms for basic research and biotech industries. The major problem was that most of the traditionally used antibiotics and resistance cassette genes cannot be used in archaeal phyla and therefore auxotrophies have to be used as selectable markers. However, nowadays very well-developed genetic toolboxes exist for the euryarchaea *Thermococcus kodakarensis, Pyrococcus furiosus, Haloferax volcanii*, and a number of methanogenic Archaea (Leigh et al., 2011). For *Pyrococcus*, it has been demonstrated that large gene clusters can be introduced for the production of several compounds (Lipscomb et al., 2014). Also for the genus *Sulfolobus*, a number of genetic systems have been established (Leigh et al., 2011). Early in the 1990s, the first transformation protocols by electroporation were established for *S. solfataricus* strains and self-transmissible vectors based on a conjugative plasmid, pNOB8, and the virus SSV1 were developed (Schleper et al., 1992; Elferink et al., 1996). The virus vector-based pMJ0503 was successfully used for the overexpression of tagged proteins in *S. solfataricus* (Albers et al., 2006). For the expression of proteins in *S. islandicus*, the plasmid pSeSD1 proved to be very useful (Peng et al., 2012). The first targeted deletion mutants were obtained in a *S. solfataricus* 98/2 PBL2025, which had a large deletion of 50 kB in the genome including many genes coding for proteins involved in sugar metabolism. As this strain was unable to grow on lactose as single carbon source, the β-galactosidase LacS could be used as marker cassette (Worthington et al., 2003). However, in this case no counterselection could be used to remove the marker cassette and therefore double deletion mutants could not be obtained. In the meantime, three model systems have developed, namely two in *S. islandicus* strains and one in *S. acidocaldarius*, which use mainly uracil auxotrophy for the selection and counterselection of mutants (She et al., 2009; Wagner et al., 2012; Zhang and Whitaker, 2012). Whereas the two *S. islandicus* strains contain a large number of transposable elements, which can lead to large genome rearrangements, the *S. acidocaldarius* genome is remarkably stable (Chen et al., 2005), which was shown by sequencing several strains isolated from North America, Russia, and Japan (Mao and Grogan, 2012). For *S. acidocaldarius* currently two uracil auxotrophic mutants are being used, MW001 (Wagner et al., 2012) and MR31 (Reilly and Grogan, 2001). For MW001 a whole set of genetic tools has been established. This includes several plasmids for the construction of markerless deletion mutants or for the insertion of tags into the genome (Wagner et al., 2012). Using these, the glucose ABC transporter of *S. solfataricus* was ectopically integrated into the MW001 genome and successfully expressed (Wagner et al., 2012). Based on the cryptic plasmid pRN1 from *S. islandicus* (Zillig et al., 1993), *Escherichia coli–Sulfolobus* shuttle vectors and expression vectors were established, which enabled the homologous or heterologous expression of tagged proteins of interest (Berkner et al., 2007, 2010). The *S. acidocaldarius* MW001 genetic system has been successfully used in a number of laboratories and helped to establish *S. acidocaldarius* as a model crenarchaeon. In a recent achievement, it was possible to harness the endogenous CRISPR/Cas system of *S. islandicus* for targeted genome editing (Li et al., 2016). This is a great next step in the direction of facilitated and accelerated manipulation of the genus *Sulfolobus*. **Table 1** gives an overview of robust and highly cited expression systems and tools for gene disruption/deletion and genomic integration for the genus *Sulfolobus.*

**Table 1:** A selection of expression systems and tools for gene disruption/deletion and genomic integration for the genus *Sulfolobus*.

Organism	Expression vectors	Gene disruption/deletion and genomic integration
*Sulfolobus acidocaldarius*	Expression plasmid pCmalLacS with a maltose inducible promoter, *lacS* marker gene, *pyrEF* selection, and amp^r^ cassette (Berkner et al., 2010)	Construction of markerless insertion and deletion mutants via double crossover based on *pyrEF*/5-FOA counterselection (Wagner et al., 2012)
*Sulfolobus solfataricus*	pSVA expression plasmid series with an arabinose inducible *araS* promoter, *pyrEF* selection, and amp^r^ cassette (Albers et al., 2006)	Gene disruption by homologous recombination via permanent insertion of the *lacS* marker gene (Albers and Driessen, 2007)
*Sulfolobus islandicus*	Expression plasmid pSeSD with a modified arabinose inducible *araS* promoter, two 6xHis tags and two protease sites for tag removal, *pyrEF* selection and an amp^r^ cassette (Peng et al., 2012)	Improved method for markerless gene deletion by combining the established *pyrEF*/5-FOA and *lacS* markers with the stringent *argD* selection (Zhang et al., 2013) Markerless gene deletion using *apt*/6-MP counterselection (Zhang et al., 2016) CRISPR-based gene knockout and integration via homologous recombination (Li et al., 2016)

*These examples represent only a fraction of the developed genetic tools, but based on their frequent usage can be considered highly reliable and successful systems. A more detailed insight into the development of genetic tools for the genus is given, for example, in a very recent review by Peng et al. (2017). *lacS*, gene coding for a β-galactosidase from *S. solfataricus* for lactose selection and blue/white screening; *pyrEF*, genes for the complementation of uracil auxotrophy; *pyrEF*/5-FOA counterselection, based on the resistance to pyrimidine analog 5-fluoroorotic acid (5-FOA) due to inactivation of the orotate phosphoribosyltransferase (*pyrE*) and orotidine 5′-phosphate decarboxylase (*pyrF*); *argD*, gene for the complementation of agmatine auxotrophy; *apt*/6-MP counterselection, based on the resistance to purine analog 6-methylpurine (6-MP) due to inactivation of a putative adenine phosphoribosyltransferase (*apt*); amp^*r*^, ampicillin resistance cassette for selection in *Escherichia coli*.*

The availability of potent genetic tools (Wagner et al., 2012; Peng et al., 2017) makes the transfer of heterologous genes to *Sulfolobus* species possible, allowing to benefit from both the metabolic diversity of *S. solfataricus* and the stability of *S. acidocaldarius*. In fact, the simpler, less promiscuous catabolism of *S. acidocaldarius* is an advantage over *S. solfataricus* in biotechnological applications, making it much easier to partly knockout metabolic pathways with the aim to redirect substrate fluxes toward a desired product.

## Untapping the Resource *Sulfolobus*

To date, extremophiles are exploited as source of thermostable enzymes, so-called extremozymes, for food and feed industry, textile and cleaning industry, pulp and paper industry, but also in scientific research and diagnostics. Starch-hydrolyzing (Elleuche and Antranikian, 2013), (hemi)cellulolytic (Beg et al., 2001; Kuhad et al., 2011), pectinolytic (Sharma et al., 2013), chitinolytic (Chavan and Deshpande, 2013), proteolytic (Li et al., 2013), and lipolytic (Hasan et al., 2006) enzymes are in high demand in industry (Elleuche et al., 2015). Enzymes of *Sulfolobus* spp. are especially interesting for such applications not only because of their great catalytic diversity, but also mainly due to their superior pH and temperature stability, which comes hand-in-hand with increased resilience toward organic solvents and resistance toward proteolysis (Daniel et al., 1982; Unsworth et al., 2007; Stepankova et al., 2013). However, also tetraether lipids, membrane vesicles with antimicrobial properties, the storage component trehalose, and novel β-galactooligosaccharides are gaining importance nowadays. The most important products are shortly described below and summarized in **Table 2**.

**Table 2:** Products and applications of *Sulfolobus* spp. reported in the literature.

Enzymes or products	Application	Citations
**Extremozymes**		
Proteases	Food, textile, and cleaning industry	Fusek et al., 1990; Hanner et al., 1990; Burlini et al., 1992; Colombo et al., 1995; Condò et al., 1998; Guagliardi et al., 2002; Gogliettino et al., 2014
Esterases/lipases	Textile and cleaning industry; synthesis of chiral fine chemicals	Suzuki et al., 2004; Park et al., 2008
Chaperonins	Biopharmaceutical protein production	Cerchia et al., 2000; Li et al., 2012
Polysaccharide degrading enzymes	Biorefinery applications for the conversion of lignocellulose into value-added products	Grogan, 1989; Moracci et al., 1995, 2000; Haseltine et al., 1996; Cannio et al., 2004; Kim et al., 2004; Kufner, 2011
**Novel biomolecules and interesting metabolites**		
Archaeal membrane components	Liposomes for drug delivery	De Rosa et al., 1994; Patel and Sprott, 1999; Krishnan et al., 2000; Benvegnu et al., 2009; Mahmoud et al., 2015
Sulfolobicins	Antibiotic agents	Prangishvili et al., 2000; O’Connor and Shand, 2002; Besse et al., 2015
Trehalose	Preservation of enzymes and drugs	Nicolaus et al., 1988; Kobayashi et al., 1996; Lernia et al., 2002
β-galactooligosaccharides	Food industry/dietary additives	Reuter et al., 1999; Petzelbauer et al., 2000

### Proteases

Stable proteases are of great interest for the industry and a vast number of different proteases from both *S. solfataricus* (Hanner et al., 1990; Burlini et al., 1992; Colombo et al., 1995; Guagliardi et al., 2002; Gogliettino et al., 2014) and *S. acidocaldarius* (Fusek et al., 1990; Lin and Tang, 1990) has been described in detail. Condò et al. (1998) described an active, chaperonin-associated aminopeptidase from *S. solfataricus* MT4. Sommaruga et al. (2014) were able to significantly improve stability and reaction yield of a well-characterized carboxypeptidase also from *S. solfataricus* MT4 by immobilizing the enzyme on magnetic nanoparticles.

### Esterases/Lipases

A serine arylesterase from *S. solfataricus* P1 was expressed. Besides its broad arylesterase activity, it was found to exhibit paraoxonase activity toward organophosphates (Park et al., 2008). With a temperature optimum of 94°C, a half-life of approximately 50 h at 90°C and high stability against detergents, urea and organic solvents, the enzyme has a high potential for industrial applications. An esterase from *S. tokodaii* strain 7 was expressed in *E. coli* and in addition to its optimal activity at 70°C remained active in a mixture of water and organic solvents such as acetonitrile and dimethyl sulfoxide (Suzuki et al., 2004).

### Chaperonins

A small heat shock protein (S.so-HSP20) from *S. solfataricus* P2 was successfully used to increase the tolerance in response to temperature shocks (50, 4°C) of *E. coli* cells (Li et al., 2012). The chaperonin Ssocpn, which requires ATP, K^+^, and Mg^2+^ but no additional proteins for its function, produced in *S. solfataricus* GΘ has been shown to yield folded and active protein from denatured materials. For this application, the chaperonin (920 kDa) was retained on an ultrafiltration cell, while the renatured substrates passed through the membrane (Cerchia et al., 2000).

### Liposomes/Membrane

The membrane of extreme thermophilic Archaea is unique in its composition due to its tetraether lipid content. Archaeal lipids are a promising source for liposomes with outstanding temperature and pH stability and tightness against solute leakage. These so-called archaeosomes are potential vehicles for drug, vaccine, and gene delivery (Patel and Sprott, 1999; Krishnan et al., 2000; Benvegnu et al., 2009; Mahmoud et al., 2015). Also the use as components for bioelectronics has been proposed (De Rosa et al., 1994; Hanford and Peeples, 2002). Unfortunately, no such applications using archaeal lipids have been published yet.

### Sulfolobicins

*Sulfolobus* spp. produce an interesting class of antibiotic proteins and peptides which are known under the term archaeocins, or more specifically sulfolobicins (Prangishvili et al., 2000; O’Connor and Shand, 2002; Besse et al., 2015). Sulfolobicins are potent and highly specific growth inhibitors targeting species closely related to the producing organism. Sulfolobicins have been identified as proteins of a size of 20 kDa in *S. islandicus* (Prangishvili et al., 2000) or heterodimers of 22 kDa per subunit in *S. acidocaldarius* (Ellen et al., 2011). They are associated with the cell membrane as well as with membrane vesicles of 50–200 nm in diameter. Known producers of sulfolobicins are *S. islandicus* strain HEN2/2 (Prangishvili et al., 2000), *S. acidocaldarius* DSM639, *S. tokodaii* strain 7, and *S. solfataricus* P2 and P1 (all strains: Ellen et al., 2011). Sulfolobicins are among the most resilient antimicrobial biomolecules withstanding temperatures of 78°C, SDS treatment, a broad pH range from 3 to 10.7, trypsin treatment, and longtime storage (Besse et al., 2015).

### Trehalose

Trehalose is crucial for anhydrobiosis in many organisms and is widely used for the preservation of enzymes and antibodies (Ohtake and Wang, 2011). On top of that it serves as a valuable chemical in the food and cosmetics industry (Richards et al., 2002). It is a known metabolite of *Sulfolobus* spp. and the biosynthetic pathways are identified (Nicolaus et al., 1988; Kobayashi et al., 1996). Since its biosynthesis is regarded to be a stress response, the selective production of trehalose is a promising target for process engineering. The enzymatic capability of *S. solfataricus* to efficiently produce trehalose was already proven by Lernia et al. (2002): In a cell-free environment, trehalose was produced from dextrins with enzymes from *S. solfataricus* MT4 in an immobilized bed reactor with a conversion rate of 90%.

### Unique Enzymes for the Synthesis of High-Value Chemicals

A number of applications for enzymes from *Sulfolobus* spp. in the synthesis of high-value chemicals have been suggested and many innovative processes have been reported: Petzelbauer et al. (2000) developed a high-temperature process for enzymatic hydrolysis of lactose for the generation of novel di- and trisaccharides (Reuter et al., 1999) using β-glycosidases from *S. solfataricus* MT4 and *Pyrococcus furiosus*. Sayer et al. (2012) characterized a thermostable transaminase from *S. solfataricus* P2. This enzyme is part of the non-phosphorylated pathway for serine synthesis which is not described in bacteria, but found in animals and plants (Walsh and Sallach, 1966; Liepman and Olsen, 2001). In *S. tokodaii*, an L-haloacid dehalogenase was found and characterized by Rye et al. (2009). This enzyme could potentially be used for the chiral production of halo-carboxylic acids which are important precursors in the fine chemical and pharmaceutical industries, as well as for bioremediation. An NAD^+^/NADH-dependent medium-chain alcohol dehydrogenase with remarkably broad substrate specificity toward primary, secondary, branched as well as cyclic alcohols and their corresponding aldehydes and ketones has been described by Raia et al. (2001). Lactonases have been described both from *S. solfataricus* MT4 (Merone et al., 2005) and from *S. islandicus* (Hiblot et al., 2012). These enzymes are attractive for biotechnological and pharmaceutical applications. An aldolase from *S. solfataricus* P1 catalyzing the reversible C-C bond formation between non-phosphorylated substrates pyruvate and glyceraldehyde to KDG was described by Buchanan et al. (1999). A sterioselective amidase from *S. solfataricus* MT4 has been described by Scotto d’Abusco et al. (2001).

## Bioprocessing with *Sulfolobus*

It is evident that *Sulfolobus* spp. accommodate a huge variety of high value-added products useful in different fields of research and industry. However, this resource has basically remained untapped until now, due to a lack of proper bioprocessing tools. Of course, many of these products can also be produced recombinantly in mesophilic hosts. Benefits of the heterologous production in mesophilic hosts are much faster growth rates, highly efficient expression, extremely well-developed process technology, and facilitated downstream processing of thermostable proteins, since a considerable amount of host cell proteins can be readily removed via heat precipitation. Nevertheless, the production of proteins difficult to express and products remaining inactive due to differences in the expression and folding machinery, call for protein production in the archaeal host (Eichler and Adams, 2005; Kim and Lee, 2006). Furthermore, certain products are native cell constituents of *Sulfolobus* spp. (e.g., archaeal membrane containing tetraether lipids), which underlines the need to generate biomass and thus of bioprocess technology.

We are convinced that thermophilic bioprocesses have the potential to compete with conventional bioprocesses, since the drawbacks of typically lower growth rates and protein expression rates can be outweighed by a number of advantages resulting from the elevated process temperature:

(1) Probably the most significant advantage is the reduced risk of contamination. Loss of complete batches or reduced productivity due to chronical basal contamination levels poses serious threats for an economically feasible bioprocess based on mesophiles (Skinner and Leathers, 2004). In case of bioprocesses with *Sulfolobus* spp. not only the high cultivation temperature, but also the low pH reduce the contamination risk.
(2) While often limited at moderate temperatures, the solubility of substrates is significantly increased at elevated process temperatures (Gray et al., 2007). This is especially crucial in applications where oligomers and polymers are used as substrates, like in waste-to-value processes based on the conversion of lignocellulosic biomass.
(3) Considering energy requirements, a further advantage over mesophilic fermentations is the reduced need for expensive, active cooling of the fermenter in large scales for the removal of excess metabolic heat. Here, high-temperature fermentations benefit from the greater difference between ambient air temperature and fermentation broth (Abdel-Banat et al., 2010).
(4) Expression systems based on so-called cold shock promoters are well known and commercialized for mesophilic hosts (e.g., the pCold expression system from Takara Bio Europe, Saint-Germain-en-Laye, France). Nevertheless, the utilization in large-scale processes is not feasible due to high costs for cooling. In high-temperature processes, cooling is much more cost-efficient due to fast heat transfer. This way, temperature-regulated expression with shifts from growth phase to production phase becomes an option.
(5) The production of volatile compounds like short-chained alcohols benefits from high process temperatures. These compounds can be continuously recovered via the off-gas stream, while no additional separation is required. Furthermore, product inhibition, a common issue when producing toxic substances like alcohols, is prevented (Zeldes et al., 2015).

Although there is a steadily growing interest in the development of extremophilic bioprocesses, no industrial process utilizing *Sulfolobus* spp. has been developed yet. Doubling times of at least 5–8 h (Brock et al., 1972; Grogan, 1989) and low biomass titers in batch cultures [max. 2 g/L dry cell weight (Schiraldi et al., 1999)] are the main obstacles for establishing efficient bioprocesses. The low biomass titer not only is a severe hindrance for biotechnological applications, but also poses a limitation for basic research because biomass and enzyme production of *Sulfolobus* spp. in shake flasks is painfully inefficient. As a result, archaeal enzymes are still mainly produced recombinantly in mesophilic hosts like *E. coli*, despite the aforementioned limitations.

In order to realize a competitive bioprocess, high cell densities in a reasonable time and economically feasible space-time yields must be achieved. This can be done by genetic engineering, optimized nutrient supply, and adjustment of process parameters. On the other hand, for bioconversion reactions, the issue of a low growth rate is not necessarily a neck-breaking drawback, if it is possible to integrate a cell-retention system combined with continuous cultivating. In that case, rather the maximum cell density, which is proportional to the volumetric catalytic activity, is a critical process parameter. However, studies on bioreactor cultivations with *Sulfolobus* spp. are still scarce.

**FIGURE 3:**
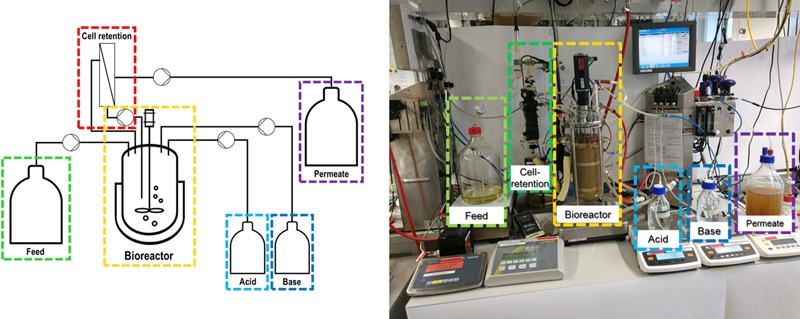
Scheme and setup of bioreactor system capable of reaching high cell densities via simultaneously applying a feed and cell-retention strategy. Nutrients can be continuously fed and at the same time spent medium containing metabolites and possibly inhibiting substances is removed via a membrane, while cells are retained.

As shown in **Table 3**, a high cell density cultivation is only reported for *S. shibatae* B12. However, it is evident that a sophisticated bioreactor setup including a cell-retention system is needed to realize a competitive bioprocess with *Sulfolobus* spp. Such a bioreactor setup is exemplarily depicted in **Figure 3**.

**Table 3:** Bioreactor cultivations with *Sulfolobus* spp. described to date.

Strain	Final biomass titer (g_DCW_/L)	Fermentation time (h)	Average volumetric productivity (g_DCW_/L/h)	Yield_X/S_ (g_DCW_/g_substrate_) and carbon sources	Cultivation mode and working volume (L)	Source
*Sulfolobus shibatae* B12 (DSM 5389)	114	358	0.32	0.156 g/g at an Yeast extract/D-glucose ratio of 1:15	Dialysis reactor, 1 L	Krahe et al., 1996
*Sulfolobus solfataricus* P2 (DSM 1617)	22.6	170	0.13	0.17 g/g at an Yeast extract/D-glucose ratio of 1:4	Constant volume fed batch, 13.8 L	Park and Lee, 1997
*Sulfolobus solfataricus* P2 (DSM 1617)	21.7	213	0.10	Yeast extract/D-glucose ratio of 1:4	Fed batch, 2.3 L	Park and Lee, 1999
*Sulfolobus solfataricus* GΘ	35	310	0.11	Yeast extract/D-glucose ratio of 1:15	Fed batch with microfiltration,10 L	Schiraldi et al., 1999
*Sulfolobus shibatae* B12 (DSM 5389)	10	200	0.05	Yeast extract/D-glucose ratio of 1:15	Fed batch, 1.3 L	Krahe et al., 1996

*DCW, dry cell weight.*

Remarkably, in none of the fermentations reported to date, defined media were used. Nevertheless, this is of high importance for the generation of platform knowledge and science-based process development. Use of defined media does allow not only the characterization and comparison of the variety of strains, but also the generation of comprehensive process understanding enabling process control and prediction. Furthermore, the use of defined media facilitates the transfer of process knowledge and speeds up process development and optimization. Another aspect worth considering is that bioprocesses that follow good manufacturing practice guidelines call for defined media to avoid batch-to-batch variability. These aspects underline the importance of the substitution of complex carbon sources, like yeast extract or protein hydrolysates, for the application of *Sulfolobus* spp. in industrial biotechnology for the production of high value-added products. Summarizing, to move *Sulfolobus* spp. into industrial biotechnology, (1) sophisticated bioreactor solutions and (2) defined media must be available.

## *Sulfolobus* as Potential Player in the Biorefinery of the Future?

Besides being a native source of high value-added products like extremozymes, extreme thermoacidophiles are predestined for the task of sustainably converting lignocellulosic biomass into value-added products due to their resilience toward harsh process conditions and their hemicellulolytic and cellulolytic properties (Turner et al., 2007). *S. solfataricus* in particular can grow on a very broad range of carbon sources (Grogan, 1989) and harbors a variety of polymer-degrading enzymes such as cellulases (Kufner, 2011), glucoamylases (Kim et al., 2004), alpha-amylases (Haseltine et al., 1996), beta-glucosidases (Moracci et al., 1995), xylanases (Cannio et al., 2004), and xylosidases (Moracci et al., 2000). Optimal growth in a hot, acidic environment means perfect synergy with the state-of-the-art method of substrate pretreatment utilizing high temperature and low pH. Although a variety of concepts for substrate pretreatment exists, the most favored process is the one of dilute sulfuric acid hydrolysis where concentrations of 0.5–1.5% sulfuric acid and temperatures between 120 and 180°C are commonly used (Carvalheiro et al., 2008; Maurya et al., 2015). Thus, pretreated substrate can be utilized in biorefinery applications based on *Sulfolobus* spp. with little to no need of neutralization and cooling of the medium. During the pretreatment process, a mixture of sugar monomers (mainly D-xylose, D-glucose, D-mannose, and L-arabinose) is released. In contrast to mesophilic hosts like *Saccharomyces cerevisiae* or *E. coli, S. acidocaldarius* lacks carbon catabolite repression (Ulas et al., 2012), thus allowing the efficient simultaneous utilization of a variety of sugars.

The combination of broad substrate specificity, lack of carbon catabolite repression, expression of polymer degrading enzymes, and extreme growth conditions make *Sulfolobus* spp. promising candidates for biorefinery applications. Following this approach, waste streams of the chemical and pulp and paper industry can be converted into value-added products. These processes would greatly benefit from the increased substrate solubility due to high temperatures and low pH. The availability of genetic tools and a broad variety of different strains are the basis for an application of *Sulfolobus* spp. in the biorefinery – however, the challenge of realizing a competitive bioprocess remains.

## Conclusion

There are several reasons to be optimistic with respect to the use of *Sulfolobus* spp. in biotechnology. Greatly reduced contamination risk, high substrate solubility, adaption to harsh substrate pretreatment conditions, facilitated removal of volatile products, and elimination of cooling costs are benefits of high-temperature processes with *Sulfolobus* spp. The genus is a source of a broad variety of temperature and acid stable enzymes as well as a producer of unique biomaterials and metabolites. A well-developed genetic toolset makes exploitation of these features possible and emergence of metabolically engineered production strains is reasonable in the near future.

However, there is still a great need for careful bioprocess development. No continuous processes are reported in the literature and sophisticated tools for monitoring and control, like on-line measurement techniques for assessing cell viability, are lacking completely. Furthermore, media development and optimization have largely been neglected. For the establishment of a competitive, long-lasting, or continuous bioprocess, it is mandatory to generate basic process knowledge to be able to understand and control the bioprocess. Thus, we will tackle this challenge to be able to add *Sulfolobus* spp. as key player in industrial biotechnology in the future.

## Author Contributions

OS conceived the idea for writing this review. JQ drafted the manuscript, while S-VA and BS contributed the chapters on genetic tools and central carbon metabolism, respectively. LS contributed the figure describing the central carbon metabolism. OS critically reviewed and corrected the manuscript and gave substantial input.

## Conflict of Interest Statement

The authors declare that the research was conducted in the absence of any commercial or financial relationships that could be construed as a potential conflict of interest.

## Supplementary Material

TABLE S1 Milestones in *Sulfolobus* research.
