# Supplementary material for: Sulfolobus – A Potential Key Organism in Future Biotechnology
Source: Front Microbiol. 2017 Dec 12;8:2474. doi: 10.3389/fmicb.2017.02474 (PMC5733018; doi:10.3389/fmicb.2017.02474)
Supplement: TABLE S1 — Milestones in Sulfolobus research. [file Table_1.DOCX]

|  | **Milestones in *Sulfolobus* research** | **Source** |
| --- | --- | --- |
| **1972**  **1975**  **1980**  **1985**  **1982**  **1989**  **1990**  **1991**  **1992**  **1993** | First mention of the term “*Sulfolobus*”  **Foundation of the genus *Sulfolobus*** and description of the species ***S. acidocaldarius*** (strain DSM 639^T^) | Brock et al. 1972 |
|  | Isolation of *Sulfolobus* strains MT-3 and MT-4 (DSM 5833). Later assigned to the species ***S. solfataricus*** *by* (Zillig et al., 1980) | De Rosa et al. 1975 |
|  | Description of the species *S. Solfataricus* and isolation of the strains ***S. solfataricus* P1** (DSM 1616^T^) and **P2** (DSM 1617) | Zillig et al. 1980 |
|  | Isolation of *Sulfolobus* strain B12. The isolate was later assigned to the species ***S. shibatae*** as type strain (DSM 5389^T^) by (Grogan et al., 1990). | Yeats et al. 1982 |
|  | Isolation of *Sulfolobus* strain 7. The isolate was later assigned to the species ***S. tokodaii*** as type strain (DSM 16993^T^) by (Suzuki et al., 2002). | Wakagi & Oshima 1985 |
|  | Fundamental work on **substrate utilization and growth inhibitors** for *S. acidocaldarius* and *S. solfataricus* strains | Grogan 1989 |
|  | Establishment of **Archaea as the** **Third Domain of Life** – The genus *Sulfolobus* was classified as member of the **phylum Crenarchaeota** | Woese et al. 1990 |
|  | Description of the species ***S. metallicus*** (DSM 6482^T^) | Huber & Stetter 1991 |
|  | **First use of electroporation** for the transfection of *S. solfataricus* with foreign DNA | Schleper et al. 1992 |
|  | ***S. solfataricus* strain 98/2** was first described in the literature | Hochstein & Stan-Lotter 1992 |
|  | Isolation of ***Sulfolobus*** species belonging to the ***islandicus*** type. Although considered a separate species no type strain has been assigned and strains are not commercially available. | Zillig et al. 1993 |

|  | **Milestones in *Sulfolobus* research (continued)** | **Source** |
| --- | --- | --- |
| **1999**  **1997**  **2001**  **2005**  **2003**  **2010**  **2012**  **2015**  **2016**  **2006**  **2007**  **2009**  **1996**  **2011**  **2017** | **Highest published cell density** of a *Sulfolobus* cultivation: Growth of *S. shibatae* B12 in a dialysis reactor yielded 114 g/L dry cell weight | Krahe et al. 1996 |
|  | Earliest developments of *Escherichia coli*-*Sulfolobus* **shuttle vectors** | Aravalli & Garrett 1997 |
|  | The species ***S. yangmingensis*** was described and strain YM1 was described as type strain (gcm BCRC 17091) | Jan et al. 1999 |
|  | **First *Sulfolobus* genome** was fully sequenced (*S. solfataricus* strain P2) | She et al. 2001 |
|  | The genome of *S. tokodaii* was sequenced | Kawarabayasi et al. 2001 |
|  | The species ***S. tengchongensis*** was described and a type strain designated (cgmcc 1.3345). | Xiang et al. 2003 |
|  | **First targeted gene disruption** in *S. solfataricus* 98/2 | Worthington et al. 2003 |
|  | Description of a **branched Entner-Doudoroff (ED)** pathway | Ahmed et al. 2005 |
|  | The genome of *S. acidocaldarius* was sequenced | Chen et al. 2005 |
|  | Revelation of **promiscuity in the semi-phosphorylative ED pathway** | Lamble et al. 2005 |
|  | Expression of heterologous genes with an **inducible promoter system** | Albers et al. 2006 |
|  | Link of oxidative **D-arabinose degradation** pathway with TCA-cycle | Brouns et al. 2006 |
|  | Detailed description of the **central carbon metabolism** of *S. solfataricus* P2 | Snijders et al. 2006 |
|  | First **stable plasmid based *E. coli* – *Sulfolobus*** spp. **shuttle vector** | Berkner et al. 2007 |
|  | The genomes of seven *S. islandicus* strains were sequenced | Reno et al. 2009 |
|  | Detailed investigation of the **metabolism of pentose sugars** | Nunn et al. 2010 |
|  | Revelation of the **absence of diauxie** during utilization of glucose and xylose by *S. acidocaldarius* | Joshua et al. 2011 |
|  | Report of a comprehensive **genome-scale metabolic model** of *S. solfataricus* | Ulas et al. 2012 |
|  | A **versatile molecular toolbox** for the manipulation of *S. acidocaldarius* was published | Wagner et al. 2012 |
|  | The genome of *Sulfolobus solfataricus* strain 98/2 was sequenced | McCarthy et al. 2015 |
|  | The **endogenous CRISPR system** was utilized for targeted genomic manipulation | Li et al. 2016 |
|  | Newly discovery of a **L-fucose degradation pathway** in *S. solfataricus* | Wolf et al. 2016 |
|  | Description of **branched-chain and aromatic amino acid catabolism** in *S. solfataricus* via Stickland reactions – a novelty in obligate aerobes | Stark et al. 2017 |

**References**

Ahmed, H., Ettema, T. J. G., Tjaden, B., Geerling, A. C. M., van der Oost, J., and Siebers, B. (2005). The semi-phosphorylative Entner–Doudoroff pathway in hyperthermophilic archaea: a re-evaluation. *Biochemical Journal* 390, 529–540. doi:10.1042/BJ20041711.

Albers, S.-V., Jonuscheit, M., Dinkelaker, S., Urich, T., Kletzin, A., Tampé, R., et al. (2006). Production of Recombinant and Tagged Proteins in the Hyperthermophilic Archaeon Sulfolobus solfataricus. *Appl. Environ. Microbiol.* 72, 102–111. doi:10.1128/AEM.72.1.102-111.2006.

Aravalli, R. N., and Garrett, R. A. (1997). Shuttle vectors for hyperthermophilic archaea. *Extremophiles* 1, 183–192. doi:10.1007/s007920050032.

Berkner, S., Grogan, D., Albers, S.-V., and Lipps, G. (2007). Small multicopy, non-integrative shuttle vectors based on the plasmid pRN1 for Sulfolobus acidocaldarius and Sulfolobus solfataricus, model organisms of the (cren-)archaea. *Nucleic Acids Res.* 35, e88. doi:10.1093/nar/gkm449.

Brock, T. D., Brock, K. M., Belly, R. T., and Weiss, R. L. (1972). Sulfolobus: A new genus of sulfur-oxidizing bacteria living at low pH and high temperature. *Archiv. Mikrobiol.* 84, 54–68. doi:10.1007/BF00408082.

Brouns, S. J. J., Walther, J., Snijders, A. P. L., van de Werken, H. J. G., Willemen, H. L. D. M., Worm, P., et al. (2006). Identification of the Missing Links in Prokaryotic Pentose Oxidation Pathways. *Journal of Biological Chemistry* 281, 27378–27388. doi:10.1074/jbc.M605549200.

Chen, L., Brugger, K., Skovgaard, M., Redder, P., She, Q., Torarinsson, E., et al. (2005). The Genome of Sulfolobus acidocaldarius, a Model Organism of the Crenarchaeota. *Journal of Bacteriology* 187, 4992–4999. doi:10.1128/JB.187.14.4992-4999.2005.

De Rosa, M., and Gambacorta, A. (1975). Extremely Thermophilic Acidophilic Bacteria Convergent with Sulfolobus Acidocaldarius. *Microbiology* 86, 156–164. doi:10.1099/00221287-86-1-156.

Grogan, D., Palm, P., and Zillig, W. (1990). Isolate B12, which harbours a virus-like element, represents a new species of the archaebacterial genus Sulfolobus, Sulfolobus shibatae, sp. nov. *Arch. Microbiol.* 154, 594–599. doi:10.1007/BF00248842.

Grogan, D. W. (1989). Phenotypic characterization of the archaebacterial genus Sulfolobus: comparison of five wild-type strains. *Journal of Bacteriology* 171, 6710–6719.

Hochstein, L. I., and Stan-Lotter, H. (1992). Purification and properties of an ATPase from Sulfolobus solfataricus. *Archives of Biochemistry and Biophysics* 295, 153–160. doi:10.1016/0003-9861(92)90501-M.

Huber, G., and Stetter, K. O. (1991). Sulfolobus metallicus, sp. nov., a Novel Strictly Chemolithoautotrophic Thermophilic Archaeal Species of Metal-Mobilizers. *Systematic and Applied Microbiology* 14, 372–378. doi:10.1016/S0723-2020(11)80312-7.

Jan, R.-L., Wu, J., Chaw, S.-M., Tsai, C.-W., and Tsen, S.-D. (1999). A novel species of thermoacidophilic archaeon, Sulfolobus yangmingensis sp. nov. *International Journal of Systematic and Evolutionary Microbiology* 49, 1809–1816.

Joshua, C. J., Dahl, R., Benke, P. I., and Keasling, J. D. (2011). Absence of Diauxie during Simultaneous Utilization of Glucose and Xylose by Sulfolobus acidocaldarius. *Journal of Bacteriology* 193, 1293–1301. doi:10.1128/JB.01219-10.

Kawarabayasi, Y., Hino, Y., Horikawa, H., Jin-no, K., Takahashi, M., Sekine, M., et al. (2001). Complete Genome Sequence of an Aerobic Thermoacidophilic Crenarchaeon, Sulfolobus tokodaii strain7. *DNA Res* 8, 123–140. doi:10.1093/dnares/8.4.123.

Krahe, M., Antranikian, G., and Märkl, H. (1996). Fermentation of extremophilic microorganisms. *FEMS Microbiology Reviews* 18, 271–285. doi:10.1111/j.1574-6976.1996.tb00243.x.

Lamble, H. J., Theodossis, A., Milburn, C. C., Taylor, G. L., Bull, S. D., Hough, D. W., et al. (2005). Promiscuity in the part-phosphorylative Entner-Doudoroff pathway of the archaeon Sulfolobus solfataricus. *FEBS letters* 579, 6865–9. doi:10.1016/j.febslet.2005.11.028.

Li, Y., Pan, S., Zhang, Y., Ren, M., Feng, M., Peng, N., et al. (2016). Harnessing Type I and Type III CRISPR-Cas systems for genome editing. *Nucl. Acids Res.* 44, e34–e34. doi:10.1093/nar/gkv1044.

McCarthy, S., Gradnigo, J., Johnson, T., Payne, S., Lipzen, A., Martin, J., et al. (2015). Complete Genome Sequence of Sulfolobus solfataricus Strain 98/2 and Evolved Derivatives. *Genome Announc.* 3, e00549-15. doi:10.1128/genomeA.00549-15.

Nunn, C. E. M., Johnsen, U., Schonheit, P., Fuhrer, T., Sauer, U., Hough, D. W., et al. (2010). Metabolism of Pentose Sugars in the Hyperthermophilic Archaea Sulfolobus solfataricus and Sulfolobus acidocaldarius. *Journal of Biological Chemistry* 285, 33701–33709. doi:10.1074/jbc.M110.146332.

Reno, M. L., Held, N. L., Fields, C. J., Burke, P. V., and Whitaker, R. J. (2009). Biogeography of the Sulfolobus islandicus pan-genome. *Proc Natl Acad Sci U S A* 106, 8605–8610. doi:10.1073/pnas.0808945106.

Schleper, C., Kubo, K., and Zillig, W. (1992). The particle SSV1 from the extremely thermophilic archaeon Sulfolobus is a virus: demonstration of infectivity and of transfection with viral DNA. *PNAS* 89, 7645–7649. doi:10.1073/pnas.89.16.7645.

She, Q., Singh, R. K., Confalonieri, F., Zivanovic, Y., Allard, G., Awayez, M. J., et al. (2001). The complete genome of the crenarchaeon Sulfolobus solfataricus P2. *Proceedings of the National Academy of Sciences* 98, 7835–7840.

Snijders, A. P., Walther, J., Peter, S., Kinnman, I., De Vos, M. G., Van de Werken, H. J., et al. (2006). Reconstruction of central carbon metabolism in Sulfolobus solfataricus using a two-dimensional gel electrophoresis map, stable isotope labelling and DNA microarray analysis. *Proteomics* 6, 1518–1529.

Stark, H., Wolf, J., Albersmeier, A., Pham, T. K., Hofmann, J. D., Siebers, B., et al. (2017). Oxidative Stickland reactions in an obligate aerobic organism - amino acid catabolism in the Crenarchaeon Sulfolobus solfataricus. *The FEBS journal*. doi:10.1111/febs.14105.

Suzuki, T., Iwasaki, T., Uzawa, T., Hara, K., Nemoto, N., Kon, T., et al. (2002). Sulfolobus tokodaii sp. nov. (f. Sulfolobus sp. strain 7), a new member of the genus Sulfolobus isolated from Beppu Hot Springs, Japan. *Extremophiles* 6, 39–44. doi:10.1007/s007920100221.

Ulas, T., Riemer, S. A., Zaparty, M., Siebers, B., and Schomburg, D. (2012). Genome-scale reconstruction and analysis of the metabolic network in the hyperthermophilic archaeon Sulfolobus solfataricus. *PLoS ONE* 7, e43401. doi:10.1371/journal.pone.0043401.

Wagner, M., van Wolferen, M., Wagner, A., Lassak, K., Meyer, B. H., Reimann, J., et al. (2012). Versatile genetic tool box for the crenarchaeote Sulfolobus acidocaldarius. *Recent advances in genomic and genetic studies in the Archaea*, 65.

Wakagi, T., and Oshima, T. (1985). Membrane-bound ATPase of a thermoacidophilic archaebacterium, Sulfolobus acidocaldarius. *Biochimica et Biophysica Acta (BBA) - Biomembranes* 817, 33–41. doi:10.1016/0005-2736(85)90065-3.

Woese, C. R., Kandler, O., and Wheelis, M. L. (1990). Towards a natural system of organisms: proposal for the domains Archaea, Bacteria, and Eucarya. *PNAS* 87, 4576–4579. doi:10.1073/pnas.87.12.4576.

Wolf, J., Stark, H., Fafenrot, K., Albersmeier, A., Pham, T. K., Muller, K. B., et al. (2016). A systems biology approach reveals major metabolic changes in the thermoacidophilic archaeon Sulfolobus solfataricus in response to the carbon source L-fucose versus D-glucose. *Molecular microbiology* 102, 882–908. doi:10.1111/mmi.13498.

Worthington, P., Hoang, V., Perez-Pomares, F., and Blum, P. (2003). Targeted Disruption of the α-Amylase Gene in the Hyperthermophilic Archaeon Sulfolobus solfataricus. *J. Bacteriol.* 185, 482–488. doi:10.1128/JB.185.2.482-488.2003.

Xiang, X., Dong, X., and Huang, L. (2003). Sulfolobus tengchongensis sp. nov., a novel thermoacidophilic archaeon isolated from a hot spring in Tengchong, China. *Extremophiles* 7, 493–498. doi:10.1007/s00792-003-0355-2.

Yeats, S., McWilliam, P., and Zillig, W. (1982). A plasmid in the archaebacterium Sulfolobus acidocaldarius. *EMBO J* 1, 1035–1038.

Zillig, W., Kletzin, A., Schleper, C., Holz, I., Janekovic, D., Hain, J., et al. (1993). Screening for Sulfolobales, their Plasmids and their Viruses in Icelandic Solfataras. *Systematic and Applied Microbiology* 16, 609–628. doi:10.1016/S0723-2020(11)80333-4.

Zillig, W., Stetter, K. O., Wunderl, S., Schulz, W., Priess, H., and Scholz, I. (1980). The Sulfolobus-“Caldariella” group: Taxonomy on the basis of the structure of DNA-dependent RNA polymerases. *Arch. Microbiol.* 125, 259–269. doi:10.1007/BF00446886.
